# Body Dissatisfaction Enhances Awareness and Facilitates the Consolidation of Body-Related Words During Rapid Serial Visual Presentation

**DOI:** 10.3389/fpsyg.2019.02614

**Published:** 2019-11-29

**Authors:** Man Yi So, Xinyu Wang, Xiao Gao

**Affiliations:** ^1^Key Laboratory of Cognition and Personality, Ministry of Education, Southwest University, Chongqing, China; ^2^Faculty of Psychology, Southwest University, Chongqing, China

**Keywords:** attentional blink, temporal attention, attentional bias, body dissatisfaction, anxiety

## Abstract

Attentional biases have received considerable focus in research on cognitive biases and body dissatisfaction (BD). However, most work has focused on spatial allocation of attention. The current two experiments employed a rapid serial visual presentation (RSVP) task to investigate attention bias to body-related words in the temporal domain among young females with high and low BD. During this task, there were two targets presented in the same stimulus stream. The first target was defined as target one (T1) and the second was defined as target 2 (T2). Participants were asked to identify T2 while ignoring T1 in single task mode or identify both targets in the dual task mode. In the current study, Experiment 1 assessed the stimulus-driven attention of body-related stimuli. Participants were required to identify a target of neutral word (T2) as quickly and accurately as possible while ignoring the preceding target (T1) of neutral, fat-, or thin-related words. As expected, we observed spontaneous attentional blink (AB) effects elicited by both fat- and thin-related T1s among participants with high BD, suggesting enhanced awareness of body-related stimuli even when this information does not have to be identified. Such effects did not emerge among participants without BD. Experimental 2 investigated the goal-directed attention of body-related stimuli, during which participants needed to identify both the T1 and neutral T2. Participants with BD showed reduced AB effects after both fat- and thin-related T1, suggesting facilitated consolidation of body-related information in goal-directed attention among participants with BD. These findings have important clinical implications that it provided insight for creating more accurate attention bias modification (ABM) task aiming at reducing and preventing BD among young females.

## Introduction

Body dissatisfaction (BD) is one of the prominent risk and maintenance factors for eating disorders (EDs; Stice and Shaw, 2002; Stice et al., 2010). BD is very common among young women across countries and cultures. For example, approximately 40–50% of women in the United States feel dissatisfaction with their body (Bearman et al., 2006). Fifty percent of adult women in Iceland (Matthiasdottir et al., 2010) and 48.3% of women in Germany (von Lengerke et al., 2012) report BD, and the proportion in China is similar to those in Western societies (Chen, 2006).

There is abundant literature on information processing preferences related to body size and shape, most of which have suggested that attentional biases toward body-related cues contribute to the etiology and maintenance of BD and EDs (Cash and Strachan, 2002; Dobson and Dozois, 2004; Lee and Shafran, 2004). Vitousek and Hollon (1990) first proposed a cognitive model of EDs to explain why and how patients with EDs developed biased cognitive processing of body-related information (Vitousek and Hollon, 1990). Later, other cognitive theories of BD suggested that schemas related to appearance, shape, and weight influence processing of body image information (Cash and Labarge, 1996; Williamson et al., 2004).

Attentional biases have received the greatest amount of focus among research on cognitive biases and BD. Early studies often used modified Stroop color naming tasks to study attentional bias in ED samples, and most of the results indicated that ED samples were often slower to name the color of disorder-relevant stimuli than the neutral cues (Perpina et al., 1998; Sackville et al., 1998; Davidson and Wright, 2002; Lee and Shafran, 2004). Later, researchers adopted the dot probe task to investigate attentional bias in EDs or BD. In this task, a pair of stimuli (e.g., a body-related stimulus and a neutral stimulus) are briefly presented on a screen, one above the other or one beside the other, and the pair of stimuli are immediately followed by a dot (the probe) in the location of one of the stimuli. Participants are required to respond to the probe as quickly and accurately as possible.

A recent literature has reviewed 22 studies exploring attention biases associated with BD (for review, see Rodgers and DuBois, 2016). It reported that studies employing a dot-probe paradigm found that when compared to individuals with low BD, individuals with high BD showed a greater attentional bias toward negative appearance-related (Rosser et al., 2010; Gao et al., 2011) as well as positive appearance-related stimuli (Glauert et al., 2010; Rosser et al., 2010; Onden-Lim et al., 2012). Studies using eye tracking measurements have reported that relative to a low BD group, women with BD tend to orient more attention toward negative appearance-related stimuli (Gao et al., 2011, 2012, 2014). However, some researchers have reported attentional avoidance of appearance-related information in the late attentional process phase (Glauert et al., 2010; Gao et al., 2012).

Several studies using various paradigms have demonstrated that attentional biases toward body-related information are associated with BD. However, most have focused on the spatial allocation of attention to body-related stimuli. Some used a dot probe paradigm (i.e., Glauert et al., 2010; Gao et al., 2011, 2012), some employed a visual search task (i.e., Smeets et al., 2011; Gao et al., 2014), while others utilized a spatial cueing paradigm (i.e., Gao et al., 2013). In all the above tasks, subcomponents of attentional bias including orientation, engagement, disengagement, and maintenance could be investigated using different experimental manipulations combining with reaction time. However, the attentional bias in the spatial domain is not the only mechanism of interest for understanding the cognitive processing of body-related information in terms of its association with BD. Below are some limitations in previous studies in this field.

First, few studies have distinguished two important components of attention, namely, goal-directed and stimulus-driven attention (Corbetta and Shulman, 2002). The former is top-down controlled attention (i.e., it focuses on relevant signals derived from task demands; Annic et al., 2016), whereas the latter is bottom-up driven attention (i.e., it is captured by salient properties of stimuli that are usually irrelevant with the task; Desimone and Duncan, 1995). The tasks used in previous studies – for example, the dot probe task and the visual search task – capture both elements of attention. However, they can’t distinguish those two components. For example, there is stimulus-driven attention in the dot-probe task, such that body-related cues could automatically capture attention among participants concerned with their body weight or shape (e.g., Gao et al., 2011). Meanwhile, there is also an element of goal-directed attention, such that participants must use attentional control to overcome the interference of body-related cues to identify the target (e.g., arrow direction or dot location).

Second, previous studies have showed that individuals with BD are more likely to provide fat-related judgments of ambiguous information (Chen and Jackson, 2005), more prone to interpret fat-related stimuli in a negative way (Chen, 2006), orient their attention quicker to body-related stimuli rather than other cues (Gao et al., 2011, 2012, 2014), and maintained more attention to fat-related stimuli (Gao et al., 2011). These findings showed increased recruitment of stimulus-driven processes, which may involve considerable attentional resources. However, previous literature focusing on the spatial domain could not determine whether the attentional orientation, engagement, or maintenance bias toward body-related information occupied more attentional resources when the location of the critical stimuli was attended. This is critical for the onset and maintenance of BD and even EDs, because excessive attention to body-related information may reflect deficits in goal-directed attentional resources, resulting in ruminating or dwelling on the negative body-related information.

Third, few studies have directly examined the attentional interference in the temporal domain among people with BD. For example, previous studies could not determine whether body-related information is more easily captured than neutral stimuli among people with BD when processing flows of information in the temporal domain. In addition, research on spatial attentional bias has reported that when body-related information is no longer present, people with BD showed delayed spatial disengagement from the location where the critical stimuli were presented (Gao et al., 2011, 2013). Thus, we might ask whether processing body-related information influences the processing of subsequent stimuli. Investigating body-related information processing in the temporal domain is critical to understanding how cognitive characteristics associated with BD contribute to dynamic alterations regarding what information reaches conscious awareness. This may then lead to a better understanding of how to intervene and prevent BD (Browning et al., 2010; Van Dam et al., 2012).

One common approach of examining the nature of human attentional processing in the temporal domain is rapid serial visual presentation (RSVP), which was first developed by Raymond (Raymond et al., 1992). In this task, stimuli appear in rapid succession in the same spatial location, at rates ranging from 8–16 stimuli per second, and the stream of stimuli typically contains 6–20 items. In the single-task mode, participants are required to identify a specific target in the distractor stream. For example, they are asked to identify a letter *X* in a numeric stream, or identify a white letter in a red-letter-stream. Detection of the target usually occurs with approximately 95% accuracy (e.g., Shapiro et al., 1997). In the dual-task mode, participants are asked to identify the first target (T1) as well as the second target (T2), which are presented in the same stimulus stream. Relative to the single task, in the dual-task mode, accuracy of T2 detection in a 100- to 500-ms window after T1 is decreased following T1 identification (e.g., Martin and Shapiro, 2008). The decrease in performance in the dual-task RSVP paradigm relative to the single-task paradigm is called an attentional blink (AB). The AB exhibits a U-shaped return to optimal T2 detection rates as the number of intervening stimuli increases (Van Dam et al., 2012).

Rapid serial visual presentation paradigms are useful for exploring the temporal mechanisms of attention as modulated by emotion (McHugo et al., 2013; for review, see Mishra et al., 2017). On the one hand, the stimulus-driven bottom-up engagement of attention could be assessed using emotional T1s, which do not have to be identified, in the single task mode. For example, the presentation of emotional T1s in a single task (for which a response is not required) hinders the identification of a subsequent T2 at short temporal lags, producing a phenomenon termed emotion-induced attentional blindness (EAB, Most et al., 2005; McHugo et al., 2013). Thus, it seems that emotional T1s automatically capture attention, occupying limited visual working memory resources, and hence compromising processing of the following T2. On the other hand, goal-directed, top-down engagement of attention could also be investigated using emotional T1s in the dual-task mode. For example, emotional T1s can increase blink magnitude (greater reduction in T2 response accuracy) at short lags compared to neutral T1s (de Jong et al., 2010; for review, see Mishra et al., 2017).

Rapid serial visual presentation has also been employed to investigate specific mechanisms of attention that are biased or impaired in a particular population (Bredemeier et al., 2011; Van Dam et al., 2012; Weierich and Treat, 2015). Previous studies have used EAB to measure the extent to which individuals with anxiety disorders exhibit increased attentional capture by – or difficulty disengaging from – concern-relevant stimuli (e.g., Olatunji et al., 2011). Other studies using symptom-related T1s in the dual-task mode observed reduced length of AB among participants with high levels of arachnophobia symptoms (Cisler et al., 2007) as well as with high levels of PTSD symptoms (Amir et al., 2009), suggesting that individuals with anxiety about spiders or PTSD symptoms may require fewer attentional resources to process symptom-related stimuli when employing goal-directed attention.

Giving the limitations of previous studies assessing the BD and attention interaction, the current two experiments employed RSVP to investigate the attentional bias to fat- and thin-related words in the temporal domain among young females with high and low BD. Experiment 1 aimed to assess the stimulus-driven attention to body-related stimuli by employing neutral, fat-, and thin-related T1s in the single-task RSVP paradigm. Participants were required to identify a neutral T2 as precisely as possible while ignoring the preceding T1. Experiment 2, by using a dual-task RSVP paradigm, sought to investigate goal-directed attention to body-related stimuli by using the same T1 as in Experiment 1. Participants needed to identify both the T1 and neutral T2 accurately. According to Vitousek and Hollon’s (1990) cognitive theory of EDs, attentional biases toward weight-related information arise as a result of underlying maladaptive schemata associated with shape, weight and self. Individuals with maladaptive schemata differ from those without in several ways, including enhanced attention to and more efficient processing of schema-related information. Based on Vitousek and Hollon’s (1990) cognitive theory of EDs and previous literature (Glauert et al., 2010; Gao et al., 2011, 2013), we expected that (1) there would be a spontaneous AB effect after both fat- and thin-related T1s in the single-task RSVP (Experiment 1) among participants with high BD. These spontaneous AB effects, on the other hand, would be weaker or non-existent among participants with low BD; and (2) compared to the low BD group, the AB effects induced by both fat- and thin-related T1s in the dual-task RSVP (Experiment 2) would be less pronounced in the high BD group, leading to a higher accuracy rate for T2s after both fat- and thin-related T1s in a short time window.

## Experiment 1

In Experiment 1, our main objective was to examine the interaction of BD and stimulus-driven attention, which may be characterized by automatic attention capture as a result of body-related stimuli among women with high BD. Single-task RSVP was employed with neutral (household words), fat-, and thin-related words as T1 and affectively neutral words as T2 (words referring to instruments). The T2 appeared anywhere from the first (lag 1) to the sixth position (lag 6) after T1. Participants were required to detect the T2 as accurately as possible while ignoring other distractors, including the T1. As described in the central capacity-limited model (Chun and Potter, 1995), the to-be-ignored T1 would receive consolidation processing and occupy considerable working memory resources if it is of high salience, which would in turn interfere with T2 identification. Thus, according to this model (Chun and Potter, 1995) and based on previous findings (Glauert et al., 2010; Gao et al., 2011, 2013), we hypothesized that women with high BD would show spontaneous AB effects in a short period after both fat- and thin-related T1s, while these AB effects would be weak or non-existent in the low BD group.

### Method

#### Participants

The sample included 57 young women drawn from undergraduate classes at Southwest University, Chongqing, China. Twenty-eight of them were in the high BD group and 29 were in the low BD group. The entire sample ranged from 18 to 25 years of age (*M* = 21.19, SD = 1.60), and their BMIs ranged from 16.23 to 28.40 (*M* = 20.06, SD = 2.31). The demographic information and self-reported data of the two groups are presented in Table 1. All were right-handed non-smokers, with no current or previous neurological or psychiatric illness, had a normal or corrected-to-normal vision, and had a normal color vision as assessed by several basic color tests.

**TABLE 1:** Demographic information of participants in Experiment 1 and Experiment 2.

	**High BD group**	**Low BD group**	***t***	***p***
**Experiment 1**				
*N*	28	29		
Age (years)	20.86 (1.84)	21.52 (1.27)	–1.58	0.120
BMI (kg/m^2^)	20.71 (2.77)	19.43 (1.59)	2.13	0.037
NPS-F	2.84 (0.27)	1.04 (0.37)	20.98	<0.001
STAI-Trait subscale	2.03 (0.49)	1.86 (0.53)	1.23	0.224
**Experiment 2**				
*N*	30	30		
Age (years)	22.60 (2.50)	22.17 (1.15)	0.86	0.392
BMI (kg/m^2^)	20.31 (2.15)	19.14 (1.52)	2.43	0.018
NPS-F	2.82 (0.27)	1.31 (0.62)	12.24	<0.001
STAI-trait subscale	2.27 (0.78)	2.01 (0.47)	1.60	0.116

*NPS-F = negative physical self scale-fatness; STAI-Trait subscale = the state-trait anxiety inventory-trait subscale.*

Following ethics approval from Southwest University, participants were recruited via on-campus advertisements. Subsequently, 338 female undergraduate students engaged in the first session of the study. After reading a general overview of the study and signing informed consent, participants completed individually administered self-reported measures of age, handedness, history of neurological or psychiatric illness, the Negative Physical Self Scale-Fatness (NPS-F), and the State-Trait Anxiety Inventory-Trait subscale (STAI-Trait subscale). Objective weight and height measurements were taken and a vision test was completed. Appointments were made with 30 women randomly selected from subgroups who scored higher than 2.5 and with 30 women who scored lower than 1.5 on the NPS-F. With the aim of obtaining extreme groups, the current cutoffs rather than the median were chosen. These cutoffs are based on previous investigations in a sample (*n* = 2152) drawn from middle schools, high schools, and colleges/universities in China ranging from 12 to 24 years of age, where the mean score on the NPS-F was 1.06 (SD = 0.90), and the mean score for females (*n* = 1242) was about 1.3 [see Figure 1 in Chen et al. (2006)]. Using these cutoffs, previous studies found robust group differences for attentional biases to body-related information (Gao et al., 2011, 2012, 2014). After completing these measures, participants took part in the experimental task session. Two participants in high BD group and 1 in the low BD group did not finish the experimental task. Thus, the final sample consisted of 57 participants.

#### Measures

##### Demographics

Participants’ age and grade in university were assessed. BMI was calculated from objectively measured height and weight [BMI = Weight (kg)/Height^2^ (m^2^)].

##### Negative physical self scale-fatness subscale (NPS-F, Chen et al., 2006)

The 11-item Fatness subscale of Negative Physical Self Scale measuring thoughts, feelings, and behaviors related to body weight dissatisfaction was used to assess BD. Each item was rated between *0* = *not at all like me* and *4* = *very much like me*. Sample items include “I am very distressed when I think about my weight,” “I am fat in others’ eyes,” and “I have tried many ways to lose weight.” Scores were obtained by summing the scores of all items and dividing this total by 11 to yield an average score ranging from 0 to 4. Higher scores reflect a higher level of dissatisfaction. The NPS-F yields internally consistent scores (α = 0.88), as well as stable scores over 3 weeks (*r* = 0.89) among female and male middle and high school students and undergraduates, and over 9 months (*r* = 0.70) among middle school and high school girls (Chen and Jackson, 2007). It has satisfactory convergent and predictive validity (Chen and Jackson, 2008) among samples of adolescents and young adults. Its alpha coefficient for the current study was α = 0.87.

##### The state-trait anxiety inventory-trait subscale (stai-trait subscale) (Smith, 1972)

STAI-Trait subscale is a brief self-rating scale assessing trait anxiety in adults. It consists of 20 items that evaluate how the respondent “generally” feels. The STAI-Trait subscale asks participants to rate the frequency of their feelings on a 4-point Likert scale ranging from *1* = *almost never* to *4* = *almost always*. Sample items include “I am a steady person,” “I lack self-confidence,” and “I feel at ease.” Scores were obtained by summing the scores of all items and dividing this total by 20 to yield an average score ranging from 1 to 4 with higher scores reflecting a higher level of trait anxiety. Its alpha coefficient for the current study was α = 0.90.

#### Stimuli

Fifty-one upright words [16 fat-related words, 16 thin-related words, 16 neutral household words, and 3 instrument-related words (*Piano*, *Flute*, and *Erhu*)] printed in white as targets and 39 inverted neutral household words printed in red as distractors were used. The 32 body related words were adapted from recent work (Gao, 2010; Gao et al., 2011). The word length and frequency were matched between the targets and the distractors. Twenty-seven female undergraduate students who did not participate in the formal experiment rated all body-related words on valence (from 1 = very negative to 9 = very positive) and arousal (from 0 = very peaceful to 8 = very excited) on 9-point Likert scales. The fat- and thin-related words were rated to differ in valence, *F*(1, 53) = 135.02, *p* < 0.001. Fat-related words (*M* = 2.14, SD = 1.15) were rated more negative than thin-related words (*M* = 5.59, SD = 1.04). Fat-related words (*M* = 4.21, SD = 1.33) and thin-related words (*M* = 4.60, SD = 1.61) showed no differences in arousal ratings, *F*(1, 53) = 0.95, *p* = 0.334. All the target words are listed in Appendix A with corresponding English translations.

#### RSVP Task

**FIGURE 1:**
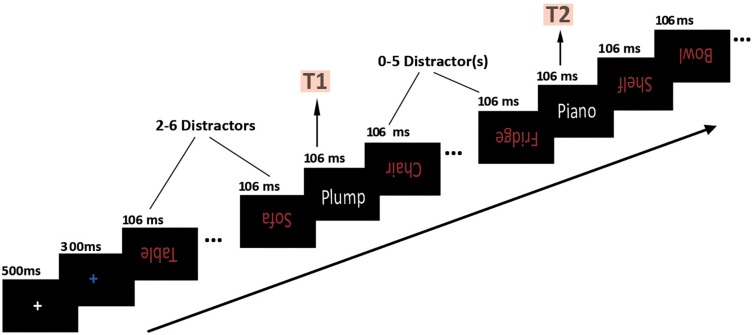
Overview of a representative experimental trial.

The RSVP procedure was programed with E-Prime 2.0 (Psychology Software Tools, Inc., Pittsburgh, PA, United States). At the beginning of the formal experiment, a white fixation and a blue fixation appeared successively in the middle of the screen and lasted for 500 and 300 ms, respectively. Shortly thereafter, 13 distractors and 2 target stimuli were displayed successively, with each visible for 106 ms and no blank interval between each other. The distractors consisted of 13 inverted neutral household words printed in red. The T1 stimulus was one of the three conditions: one of (1) 16 fat-related words, (2) 16 thin-related words, or (3) 16 neutral household words. The T2 stimulus was one of the three instrument words. Each T1 and T2 stimulus was presented in an upright position and printed in white, with the same probability of occurrence. The T1 appeared randomly and equiprobably in the third to seventh position in the stimuli series, and the T2 appeared randomly and equiprobably from the first (lag 1) to the sixth (lag 6) position after T1. Other positions aside from T1 and T2 were filled with distractors. All distractors and targets were displayed in the center of the black background. This task was illustrated in Figure 1.

In single-task RSVP, participants were presented with a question about T2 (“Please identify the first upright word: Piano, Flute, Erhu, or I don’t know”), while ignoring T1. The corresponding response key for each answer was counterbalanced across participants. In this task, participants were required to make judgments on T2 as accurately as possible. The question appeared until a response was made or for 5000 ms maximum. Participants would be led into the next trial after a 500-ms interval during which the screen remained black and blank.

#### Procedures

Before the experiment, participants were told that the current study aimed to determine human’s visual reaction speed and would last for about 20 min. Each participant completed a total of 648 trials of 18 conditions (three categories of T1 × 6 T1–T2 lags), which were randomly and equally presented in six blocks. Thus, each block consisted of 108 trials with each condition having six trials.

Individual testing sessions were scheduled between 8:30 and 11:30 am and 2:00–5:30 pm in a quiet soundproof room. Following the session, participants were debriefed about the research purposes and paid 30 Yuan as compensation for their time.

#### Data Analysis

T2 percent accuracy was analyzed using a 2 (Group: BD vs. Control) × 3 (T1 category: Fat, Thin, and Neutral words) × 6 (Lag: Lag 1–6) mixed model ANOVA. Then, for each category of T1, correlations were calculated between overall T2 percent accuracy and T2 percent accuracy at each lag with NPS-F and STAI-Trait subscale scores, respectively. *Post hoc* comparisons for significant main effects and interactions, as well as correlational analysis, used a Bonferroni correction to optimize power while minimizing Type I error rate. All statistics were computed using IBM SPSS Statistics 21.0.

### Results

#### Influence of BD on T2 Identification

A 2 (Group: BD vs. Control) × 3 (T1 category: Fat, Thin, and Neutral words) × 6 (Lag: Lag 1–6) mixed model ANOVA on T2 percent accuracy revealed a significant main effect of Lag, *F*(5, 275) = 20.25, *p* < 0.001, partial η^2^ = 0.27, with lower T2 accuracy at Lags 1 and 2 than at Lags 3–6. The main effect of Group was also significant, *F*(1, 55) = 19.63, *p* < 0.001, partial η^2^ = 0.26, reflecting better overall performance in the Control group (*M* = 0.94, *SE* = 0.005) as compared to the high BD group (*M* = 0.91, *SE* = 0.005). There were also significant interactions for Group × Lag, *F*(5, 275) = 10.24, *p* < 0.001, partial η^2^ = 0.16; Group × T1 category, *F*(2, 110) = 3.98, *p* = 0.023, partial η^2^ = 0.07; Lag × T1 category, *F*(10, 550) = 3.43, *p* = 0.001, partial η^2^ = 0.06; as well as a three-way interaction of Group × T1 × Lag, *F*(10, 150) = 2.83, *p* = 0.007, partial η^2^ = 0.05.

**FIGURE 2:**
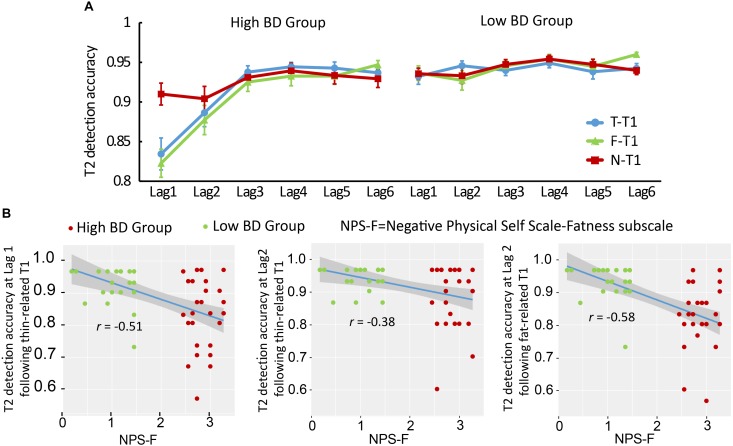
Mean percentage of correct T2 identifications and correlation analysis in single task RSVP, Experiment 1. **(A)** Mean percentage of correct T2 identifications as a function of Tl category and the lag between Tl and T2. Error bars represent the standard error of the mean. **(B)** Correlation analysis between NPS-F scores and percentage of correct T2 identifications at Lag 1 following fat-related Tl, as well as at Lag 1 and Lag 2 following thin-related Tl, with 95% confidence interval presented.

Simple effect analysis was conducted based on the three-way interaction. Spontaneous AB effects were observed only in the high BD group when the T1s were fat- and thin-related words. T2 detection was significantly lower at Lags 1 and 2 than at Lags 3–6 following both fat- and thin-related T1s (all *p*s < 0.001; Figure 2) in the high BD group. The low BD group did not show such AB effects. Meanwhile, the neutral T1 also elicited AB effects in the high BD group, such that T2 detection was worse at Lag 2 than at Lags 3–5 (all *p*s < 0.031). Comparisons between different T1 categories at different lags in the high BD group showed that T2 detection accuracy at Lag 1 after both fat- and thin-related T1s was lower as compared to those after neutral T1s (all *p*s < 0.001), indicating that in the high BD group, the magnitudes of AB effects elicited by fat- and thin-related T1s were higher than were those elicited by neutral T1s at Lag 1. The difference disappeared at later lags. However, there were no such spontaneous AB effects in the Control group (all *p*s > 0.099). When comparing T2 detection between the two groups, T2 detection accuracy at Lags 1 and 2 after both fat- and thin-related T1s was significantly lower in the high BD group than it was in the Control group (all *p*s > 0.024). No such difference was observed after neutral T1s.

#### Correlation Between BD and T2 Identification

Correlational analyses were computed across the two groups (*n* = 57). Using the Bonferroni correction, correlations were considered significant if the *p*-value was <0.05/18 = 0.003. NPS-F scores negatively correlated with T2 detection at Lag 1 following fat-related T1s (*r* = −0.58, *p* < 0.001), and T2 detection at both Lag 1 (*r* = −0.51, *p* < 0.001) and Lag 2 (*r* = −0.38, *p* = 0.003) following thin-related T1s (Figure 2B). Other correlations were not significant. STAI-Trait subscale scores did not correlate with T2 percent accuracy in any condition (all *p*s > 0.05).

### Discussion

Experiment 1 investigated stimulus-driven attention to body-related stimuli by employing neutral, fat-, and thin-related T1 in a single-task RSVP paradigm. Participants were required to identify neutral T2s as accurately as possible while ignoring T1s. As expected, we observed spontaneous AB effects elicited by both fat- and thin-related T1s among participants with high BD, suggesting that body-related words capture their attention automatically even when the information is task-irrelevant. These effects did not emerge among participants without BD. When comparing the performance of the two groups in T2 identification, high BD group showed lower T2 response accuracy following both fat- and thin-related T1 during short temporal lags (106–212 ms). The mere presentation of body-related words produced a deficit in awareness of subsequent neutral stimuli at short temporal lags in the high BD group, showing an enhancement in stimulus-driven attention. These findings in the high BD group replicated observations from previous studies, where emotional stimuli were presented as to-be-ignored T1s in the RSVP stream while participants searched for a single target (Most et al., 2005; Mathewson et al., 2008; McHugo et al., 2013). The enhancement in stimulus-driven attention to both fat- and thin-related T1s in the high BD group is consistent with previous studies focusing on the spatial domain reporting speeded detection of body-related words (Gao et al., 2011) or pictures (Gao et al., 2012) among women with BD.

## Experiment 2

As described in the Introduction, Experiment 2 aimed to investigate goal-directed attention to fat- and thin-related words in a dual-task RSVP paradigm. Participants were required to identify both T1 and T2 words as accurately as possible in an RSVP stream. Neutral, fat-, and thin-related words were again used as the three T1 categories, while neutral instrument words served as T2 words.

### Method

Sixty young women drawn from undergraduate classes at Southwest University, Chongqing, China, 30 of whom were in the high BD group and 30 in the low BD group. The entire sample ranged from 18 to 25 years of age (*M* = 21.54, SD = 2.16), and their BMIs ranged from 16.02 to 26.17 (*M* = 19.73, SD = 1.99). All were right-handed non-smokers, with no current or previous neurological or psychiatric illness, had a normal or corrected-to-normal vision, and had a normal color vision as assessed by several basic color tests. The demographic information and self-reported data of participants are presented in Table 1.

The methods of Experiment 2 were the same as in Experiment 1 with one modification. Experiment 2 used a dual-task RSVP paradigm in which participants were presented with a question about the T1 (“Please identify the second upright word: fat body word, thin body word, household word, or I don’t know”) and a question about the T2 (“Please identify the first upright word, Piano, Flute, Erhu, or I don’t know”). The corresponding response key for each answer was counterbalanced across participants. As in the single-task RSVP in Experiment 1, each participant completed a total of 648 trials of 18 conditions (three categories of T1 × 6 T1–T2 lags), which were randomly and equally presented in six blocks. Thus, each block consisted of 108 trials with each condition having six trials.

T1 detection accuracy was analyzed using two-way repeated measures ANOVAs (T1 category × Group). In order to investigate the influence of BD on T2 detection, T2 percent accuracy was analyzed using a 2 (Group: BD vs. Control) × 3 (T1 category: Fatness, Thinness vs Neutral words) × 6 (Lag: Lag 1–6) mixed model ANOVA. In the dual-task RSVP, an individual’s mean percentage accuracy for T2 words was calculated for trials where the T1 was identified correctly (Rokke et al., 2002; Fox et al., 2005). Then, for each T1 category, correlations were computed between overall T2 percent accuracy and T2 percent accuracy at each lag with NPS-F and STAI-Trait subscale scores, respectively. *Post hoc* comparisons for significant main effects and interactions, as well as correlational analysis, was conducted using the Bonferroni correction. For all analyses, *p*-values were corrected for deviations according to the Greenhouse–Geisser correction (Greenhouse and Geisser, 1959).

### Results

#### Influence of BD on Identification of Different T1s

A 2 (Group: BD vs. Control group) × 3 (T1 category: Fat, Thin, and Neutral words) ANOVA on correct T1 identification rates showed no significant main effect of Group, *F*(1, 58) = 0.594, *p* = 0.444, partial η^2^ = 0.01; T1 category, *F*(2, 116) = 0.93, *p* = 0.368, partial η^2^ = 0.02; or their interaction, *F*(2, 116) = 1.29, *p* = 0.272, partial η^2^ = 0.02.

#### Influence of BD on Correct T2 Identification

**FIGURE 3:**
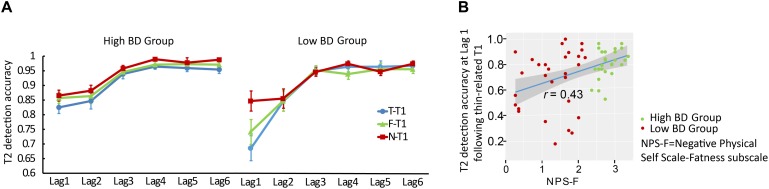
Mean percentage of correct T2 identifications and correlation analysis in dual task RSVP, Experiment 2. **(A)** Mean percentage of correct T2 identifications as a function of Tl category and the lag between Tl and T2. Error bars represent the standard error of the mean. **(B)** Correlation analysis between NPS-F scores and percentage of correct T2 identifications at Lag 1 following thin-related Tl, with 95% confidence interval presented.

A 2 (Group: BD vs. Control group) × 3 (T1 category: Fat, Thin, and Neutral words) × 6 (Lag: Lag1 – Lag6) mixed model ANOVA on T2 percent accuracy revealed a main effect of Lag, *F*(5, 285) = 50.38, *p* < 0.001, partial η^2^ = 0.77, with lower T2 accuracy at Lags 1 and 2 than at Lags 3–6 (correct identification of the T2 was about 80% for Lag 1, 86% for Lag 2, 95% for Lag 3, 96% for Lag 4 and Lag 5, and 97% for Lag 6). Further, the main effect of T1 category was significant, *F*(2, 114) = 9.06, *p* < 0.001, partial η^2^ = 0.14. *Post hoc* analysis using the Bonferroni correction indicated that participants were generally more accurate in detecting T2 words after neutral T1s (*M* = 0.93, SE = 0.07) than after fat-related T1s (*M* = 0.92, SE = 0.01; *p* = 0.029) or thin-related T1s (*M* = 0.91, SE = 0.01; *p* = 0.001) but did not differ significantly between fat- and thin-related T1s (*p* = 0.221). There was no significant main effect of Group, *F*(1, 57) = 1.11, *p* = 0.296, partial η^2^ = 0.02. Notably, the effect of T1 category on T2 accuracy varied as a function of Lag, *F*(10, 570) = 5.28, *p* < 0.001, partial η2 = 0.09, and this pattern was different between the two groups, as evidenced by the significant Group × T1 category × Lag interaction, *F*(10, 570) = 3.08, *p* = 0.004, partial η^2^ = 0.05 (Figures 2A, 3A).

For a proper interpretation of the three-way interaction, we tested the lag contrasts for each of the T1s separately within each group. For both groups, detection of T2 words after each T1 category was impaired at Lag 1 (all *p*s < 0.001) and Lag 2 (all *p*s < 0.016), while these AB effects rebounded beginning at Lag 3. For the low BD group’s performance at Lag 1, participants were less accurate in identifying T2 words after thin-related T1s (*M* = 0.70, SE = 0.03; *p* < 0.001) and fat-related T1s (*M* = 0.75, SE = 0.03; *p* < 0.001) than after neutral T1s (*M* = 0.86, SE = 0.03), while the high BD group did not show a significant difference in T2 identification accuracy between the three T1 categories. Meanwhile, for Lag 1, the low BD group showed lower accuracy in identifying T2 following thin-related (*p* = 0.007) and fat-related T1 words (*p* = 0.028) as compared to the high BD group (for thin-related T1: *M* = 0.83, SE = 0.03; for fat-related T1: *M* = 0.86, SE = 0.03). No other significant simple effects were observed.

#### Correlation Between BD and T2 Identification

Correlational analyses were computed across two groups (*n* = 60). Using the Bonferroni correction, correlations were considered significant if the *p*-value was < 0.05/18 = 0.003. NPS-F scores positively correlated with T2 identification accuracy at Lag 1 following thin-related T1 words (*r* = 0.43, *p* < 0.001, Table 2, and Figure 3B). Other correlations were not significant. STAI-Trait subscale scores did not correlate with T2 percent accuracy in any condition (all *p*s > 0.05).

**TABLE 2:** Correlation matrix between NPS-F and T2 response accuracy rate in Experiment 1 and Experiment 2.

	**T-Lag 1**	**N-Lag 1**	**F-Lag 1**	**T-Lag 2**	**N-Lag 2**	**F-Lag 2**
**Experiment 1**						
NPS-F	−0.51*^*a*^*^∗∗∗^	−0.21	−0.58*^*a*^*^∗∗∗^	−0.38*^*a*^*^∗∗^	−0.22	−0.29^∗^
**Experiment 2**						
NPS-F	0.43*^*a*^*^∗∗∗^	0.06	0.24	0.04	0.11	0.05

*NPS-F = negative physical self scale-fatness; STAI-Trait subscale = the state-trait anxiety inventory-trait subscale; T = thin-related T1; N = neutral T1; F = fat-related T1. *a* significant after Bonferroni correction. ^∗^*p* < 0.05, ^∗∗^*p* < 0.01, and ^∗∗∗^*p* < 0.001.*

### Discussion

Experiment 2 investigated the goal-directed attention of body-related stimuli by employing neutral, fat-, and thin-related words as T1s and neutral words as T2s in a dual-task RSVP paradigm. Participants were required to identify both the T1 and the following T2 as accurately as possible. Significant AB effects were observed among both groups, such that correct detection of T2 after each T1 category was impaired at Lag 1. Notably, the magnitudes of AB effects following fat- and thin-related T1s were significantly smaller in the high BD group than they were in the low BD group, indicating more effective processing of body-related words in goal-directed attention. These observations are consistent with previous findings in studies using RSVP tasks among participants with anxiety.

## General Discussion

The present two experiments, employing single- (Experiment 1) and dual-task RSVP (Experiment 2), investigated the attentional interference of fat- and thin-related words in the temporal domain. As expected, participants with BD showed stimulus-driven processing in body-related information. Specifically, in the single-task RSVP (Experiment 1), participants with BD showed spontaneous AB effects after both fat- and thin-related T1 words. No such effect emerged in the low BD group, suggesting that body-related words automatically capture attention in the high BD group, even when these words were to-be-ignored stimuli. In the dual-task RSVP (Experiment 2), participants in the low BD group had increased magnitudes of AB effects following body-related T1 words as compared to neutral T1 words. Participants with BD did not show this difference. The low BD group showed lower accuracy in identifying T2 words at Lag 1 following both fat- and thin-related T1 words as compared to high BD group, indicating reduced AB effects after both fat- and thin-related T1 words among BD group. The findings from Experiment 2 suggest that participants with BD showed more effective goal-directed attention to body-related information.

### Attentional Blink When Body-Related Information Does Not Have to Be Identified

Experiment 1 investigated stimulus-driven attention to body-related stimuli by employing neutral, fat-, and thin-related T1 in a single-task RSVP paradigm. Participants were required to identify neutral T2s as accurately as possible while ignoring T1s. The observation in Experiment 1 supported the first hypothesis that high BD group showed a spontaneous AB effect after both fat- and thin-related T1s in the single-task RSVP. Whereas, low BD group did not show such AB effects. These observations indicate that participants with high BD show enhanced stimulus-driven attention to both fat- and thin-related stimuli in the temporal domain, which has also been observed in previous studies using different paradigms. For example, participants with high BD tend to orient their attention more frequently and more quickly to body-related information as compared to neutral information (Gao et al., 2011). They also allocated more attentional resources to body-related information relative to neutral information (Gao et al., 2014), and perceive vigorous information as negative body-related information (Chen and Jackson, 2005). Correlational analyses found that T2 detection in the short interval after fat- and thin-related T1 words were only negatively correlated with BD, but not anxiety. These observations are particularly important, as they may suggest that alterations in rapid processing of body-related information may be related to BD itself, and not necessarily just anxiety.

One explanation may be that the to-be-ignored body-related information automatically captures attention among individuals with BD, thereby initiate bottom-up processing and resulting in more attentional resources allocated to its consolidation and identification. This would then leave fewer resources available for neutral T2 identification. Results from event-related potential (ERP) studies on emotion-induced blindness gave support to this inference. One previous study reported that larger ABs induced by (to-be-ignored) emotional T1s were associated with an enhanced early posterior negativity (EPN, around 200 ms) and late positive potential (LPP, 300–800 ms) ERP component (MacLeod et al., 2017). The EPN has been suggested to represent early semantic activation and selection for further processing in working memory, while the LPP has been suggested to represent the consolidation of a stimulus in working memory (Kennedy and Most, 2015). Future ERP studies are needed to investigate the neutral pattern underlying the current observations in the high BD group.

It is worth noting that the word stimuli in the current study were rated on valence and arousal before the formal experiments. Fat-related words were rated as negative while thin-related words were rated as positive, and both of them were rated as moderate in arousal. However, the low BD group did not show a spontaneous AB effect after body-related T1 words, which differs from previous observations showing that high-arousal or emotional T1 words result in ABs of 200–500 ms, even when they did not need to be identified (for review, see Mishra et al., 2017). These findings may suggest that for people without BD, body shape-related information may be processed differently from emotional stimuli (e.g., fearful, disgusting or happy) that trigger our basic emotions, even though body-related words in the current study were rated as positive or negative with moderate arousal.

### Attentional Processing of Body-Related Information When It Has to Be Identified

In Experiment 2, participants were required to identify both T1 and T2 words. Fat-, thin-, and neutral words were employed as T1s and neutral words were utilized as T2s. There was no significant difference in T1 identification regardless of category or the extent of BD.

Consistent with previous studies, we observed poorer performance in T2 identification in a short period after fat- and thin-related T1s as compared to neutral T1s in the low BD group. These observations could be explained, in part, by the two-stage central capacity-limited model (bottleneck theory) of AB (Chun and Potter, 1995). According to this model, processing a target to the level of identification requires two discrete stages: (1) processing and representing of stimulus features (e.g., the perceptual/semantic categorization), which is temporary and fragile, and (2) sustained attention, which is a capacity-limited consolidation stage in working memory and can result in consolidation of stimulus identity sufficient for recognition or report. All the targets in the RSVP stream are encoded and processed up to stage 1. The AB effects happen during the consolidation stage of T1 when there are insufficient attentional resources for T2 identification. Usually, the duration required for T1 consolidation is thought to be around 200–500 ms. Thus, findings from the low BD group suggest that the consolidation stages of fat- and thin-related T1 words recruited more attentional resources than did those of neutral T1s. This would then result in poorer performance on T2 identification in short lags. These enhanced AB effects happened around 106 ms after a body-related T1 appeared, and it rebounded at Lag 2 around 212 ms.

High BD group, who also showed significant AB effects in the dual-task RSVP, did not show such difference in AB magnitudes after fat- or thin-related T1 as compared to those after neutral T1s. The magnitudes of AB effects following fat- and thin-related T1 words in the high BD group were significantly smaller than those in the low BD group. These findings are consistent with previous studies investigating temporal processing of emotional information in anxiety (Arend and Botella, 2002; Cisler et al., 2007) and PTSD (Amir et al., 2009). It has been suggested that people with anxiety or PTSD may process disorder-relevant stimuli more rapidly and efficiently as compared to neutral information, resulting in faster recovery of attentional resources after encountering threatening information (Amir et al., 2009). One explanation regarding the reduced AB effects following body-related T1 words in high BD group may be that both fat- and thin-related words trigger body-related anxiety or concerns among people with BD. Attentional control processing may be involved to cope with the negative emotion, and thus the consolidation stages for body-related information identification may be shortened implicitly or automatically. These processes among BD group may involve attentional valiance to body-related information in the first stage as well as inhibition of processing that information in the second stage.

### BD, EDs, and Anxiety

The current findings have important clinical implications for understanding body image disturbance or anoxia nervosa (AN). Specifically, the findings among women with BD were consistent with previous observations in participants with high anxiety. For example, Barnard et al. (2005) found that threat-related distractors automatically captured the attention of high-anxiety participants and interfered with the following target identification. Arend and Botella (2002) as well as Lystad et al. (2009) manipulated the valence of T1 words and reported that T2 identification accuracy improved following anxiety-related T1s. In these studies, high-anxiety participants enhanced awareness of threat-related stimuli even when this information does not have to be identified, which reduces the accuracy of the following target. Nevertheless, in goal-directed attention, the facilitated consolidation of anxiety-related information among participants with anxiety. Thus, performance on T2 identification in both single- and dual-task RSVP among participants with high BD or anxiety showed a similar response pattern. Specifically, participants with high BD or anxiety automatically attended to critical stimuli (body- or anxiety-related stimuli) when the stimuli were designated as distractors, and they processed these stimuli more efficiently than they did neutral targets when the stimuli were employed as targets to identify. On the contrary, the opposite pattern was observed in depression, such that greater AB effects following negative T1s were observed among participants with high depression scores (Koster et al., 2009) or when participants were in a sad mood as compared to a happy mood (Rokke and Lystad, 2015). Likewise, studies focusing on the spatial domain of visual attention have reported similar patterns of attentional bias for BD, EDs, and anxiety disorders (Rieger et al., 1998; Amir et al., 2003; Lee and Shafran, 2004; Gao et al., 2013).

Several investigations have reported high overlap in co-morbidity between anxiety and EDs. One influential study (Lilenfeld et al., 1998) using contemporary family-epidemiological methods to examine patterns of comorbidity and family aggregation of psychiatry disorders for AN and bulimia nervosa (BN) showed that nearly every AN proband, more than two thirds of BN probands, and almost half of their relatives had a lifetime history of an anxiety disorder. On the other hand, lifetime rates of major depression disorders were in the low to middle range of those previously reported ED probands and their first-degree relatives. Depression and EDs, thus, appear to be transmitted independently in families. Abundant later work has consistently shown that BD is associated with concurrent overall anxiety symptoms and symptoms of generalized anxiety disorder, panic disorder, social anxiety disorder, and separation anxiety disorder in community samples (Touchette et al., 2011; Cruz-Sáez et al., 2015; Dooley et al., 2015; Duchesne et al., 2017; Vannucci and Ohannessian, 2017).

The above observations suggest that BD and anxiety may interact tightly with each other, and they may also have very similar visual attentional processing to disturbance-related information. Brain imaging studies have provided evidence to support this inference. The limbic system (especially amygdala and insula) and prefrontal cortex (PFC; medial PFC, and ventrolateral PFC in particular), which is involved in the processing of threat-based stimuli, was found to be actively involved when processing threat-related information among participants with anxiety (Monk et al., 2006, 2008), watching body-related information that could elicit BD among healthy young women (Friederich et al., 2010), among overweight young girls (Gao et al., 2016), when reading negative body-related words among patients with AN (Miyake et al., 2010), as well as when viewing distorted images of themselves among patients with AN (Seeger et al., 2002). Therefore, the cognitive models explaining how attentional bias operates in anxiety could map well onto BD/EDs and also may suggest new ideas on how and why attentional bias might develop and function within the context of BD/EDs (for reviews, see Cisler and Koster, 2010; Aspen et al., 2013).

As people with high BD as well as those with high anxiety show similar attentional processing patterns to disturbance-related information, interventions – namely, attention bias modification (ABM) – aimed at reducing attentional bias and in turn reducing anxiety symptoms among patients with anxiety (MacLeod et al., 2002; Hakamata et al., 2010) should be useful in reducing attentional bias and BD among participants with BD or patients with EDs. Indeed, two studies have used the principles of ABM among healthy undergraduate women (Smith and Rieger, 2006; Smeets et al., 2011). However, no consistent observations have emerged yet, and the effects of ABM in reducing BD appear to depend on the extent of participants’ BD. Thus, in the future, it is critical to address whether ABM can reduce BD or increase body esteem via reducing maladaptive attentional bias toward body-related information, and on the other hand, whether participants could benefit from ABM via strengthening adaptive attentional processing of body-related information. Therefore, future studies are warranted to address these questions in diverse populations (e.g., participants with BD, patients with a different kind of EDs).

### Limitations and Future Directions

There were some limitations in the current study. First, this is the first study investigating the moderating effects of BD on body-related stimuli processing in the temporal domain. Therefore, future studies are warranted to reveal the underlying mechanism. For example, by manipulating body-related stimuli as T1, T2, or the background distractors in the RSVP paradigm, it is possible to answer the following questions: (1) whether BD associates with prolonged attention to representations of now-absent visual information and (2) whether people with BD have a failure to inhibit encoding of non-task body-relevant stimuli. Second, the current sample size was relatively small. Thus, generalization to clinical samples should be undertaken with caution, and the findings should be replicated in larger and more diverse samples. Finally, the fat- and thin-related words used in this study differed both in the content and in the valence. Future research is needed to separate the effects of the content and the valence of the stimuli on the attentional processing.

## Conclusion

In conclusion, by employing single- and dual-task RSVP, we provide evidence that BD is associated with marked differences in the temporal mechanisms of perceptual processing of body- relevant information. Young females with BD exhibit robustly enhanced awareness of body-related stimuli, indicating increased recruitment of stimulus-driven processes. In the meantime, BD is associated with facilitated consolidation of body-related information, suggesting more effective processing of body-related information in goal-directed processing.

## Data Availability Statement

The datasets generated for this study are available on request to the corresponding author.

## Ethics Statement

The studies involving human participants were reviewed and approved by the Ethics Committee of the Psychology Department of Southwest University. The patients/participants provided their written informed consent to participate in this study.

## Author Contributions

MS drafted the manuscript. XW analyzed the data and edited the manuscript. XG designed the study and collected the data.

## Conflict of Interest

The authors declare that the research was conducted in the absence of any commercial or financial relationships that could be construed as a potential conflict of interest.
